# From field to sky: measurement and modeling of transgenic switchgrass pollen dispersal in the atmosphere

**DOI:** 10.1007/s10661-026-15632-3

**Published:** 2026-08-03

**Authors:** Manu Nimmala, Hope A. Gruszewski, Regina Hanlon, Landon Bilyeu, Tyler Newton, Jessica Stockdale, Reginald J. Millwood, Charles Neal Stewart Jr., Craig W. Powers, Shane D. Ross, Hosein Foroutan, David G. Schmale III

**Affiliations:** 1 Mechanical Engineering, Virginia Tech, Blacksburg, VA 24061 USA; 2 School of Plant and Environmental Sciences, Virginia Tech, Blacksburg, VA 24061 USA; 3 Aerospace and Ocean Engineering, Virginia Tech, Blacksburg, VA 24061 USA; 4 Civil and Environmental Engineering, Virginia Tech, Blacksburg, VA 24061 USA; 5 Department of Plant Sciences, University of Tennessee, Knoxville, TN 37996 USA

**Keywords:** Switchgrass, Transgenic, Pollen dispersal, Bioaerosol, Drone sampling, Pollen transport, Fluorescence, Genetically Engineered Crop

## Abstract

Accurate tracking and measurement of pollen dispersal in the atmosphere are essential for assessing cross-pollination risks, particularly in the case of genetically engineered (GE) crops. We conducted a series of unique release-recapture field studies with GE switchgrass in Oliver Springs, TN, USA. Two hundred transgenic switchgrass plants (*Panicum virgatum L.* “Performer”) were planted at the center of a clear-cut field, with one block of 100 plants expressing orange fluorescent protein (OFP) under a switchgrass ubiquitin promoter (PvUBI1) and another block of 100 plants expressing OFP driven by a maize pollen-specific promoter (Zm13). Pollen was sampled from the atmosphere using fixed (ground-based) and mobile (drone-based) sampling devices at different distances from the source field, with Lagrangian stochastic dispersal simulations run for sampling periods using high-resolution wind measurements. The pollen emission rate was estimated by combining simulated and measured pollen concentrations, and strong diurnal trends were observed. Diurnal emission rate trends were positively correlated with wind speed, temperature, and vapor pressure deficit, while negatively correlated with relative humidity. In low-wind meandering conditions, incorporating changing wind direction into the dispersal modeling improved pollen emission rate estimation and model-measurement comparisons. This study assesses the effectiveness of high- and low-volume pollen samplers in relation to source strength up to 1 km from the source, enhancing understanding of pollen measurement techniques. Additionally, it is a proof-of-concept for drone-based pollen sampling and GMO pollen tracking using fluorescence measurements. Results from our experiments have significant implications for cross-pollination risk assessment, prediction, and management of airborne allergens.

## Introduction

Accurate tracking and measurement of pollen dispersal in the atmosphere are important for assessing cross-pollination risks (Aylor et al., 2003; Nimmala et al., 2024), particularly in the case of genetically engineered (GE) crops. Beyond biosecurity, pollen dispersal measurements are also critical for allergen management, where timely and accurate reporting of airborne concentrations have direct public health implications (Beggs, 2024; Tummon et al., 2021). Wind-dispersed pollen is the primary method of gene flow in many grasses, including switchgrass (*Panicum virgatum*), an important bioenergy crop (Sofiev & Bergmann, 2012). It is a perennial, warm-season C4 bunchgrass found across most of eastern North America—from northern Mexico to southern Canada. Originally adopted as a forage crop, it is now a leading candidate for large-scale lignocellulosic biofuel feedstock in the USA and beyond (Parrish & Fike, 2005). There is increasing concern that the rapid growth and development of switchgrass as a biofuel could result in gene flow from GE switchgrass fields to nontransgenic fields (including wild populations), leading to both financial and ecological damage (Ahrens et al., 2011; Ecker et al., 2015; Kwit & Stewart, 2012; Millwood et al., 2017; Stockdale & Millwood, 2023). These changes could be compounded by the effects of extreme weather, where rising temperatures may result in altered native switchgrass territory (Ahrens et al., 2014). Therefore, there is an urgent need for field experiments and modeling efforts to characterize the dispersal of airborne switchgrass pollen in relation to meteorological factors for regulation and risk management purposes.

There are limited experimental and modeling studies on switchgrass gene flow (Kwit & Stewart, 2012); these model pollen dispersal with and without wind breaks (Auer et al., 2016), experimentally quantify the dispersal and cross-pollination of transgenic switchgrass (Millwood et al., 2017), and model transport in low and high-wind conditions (Ecker et al., 2013). In 2011, Millwood and colleagues conducted the first regulated transgenic switchgrass field experiments in the USA (Millwood et al., 2017). A 3-year field experiment was performed in Oliver Springs, TN, USA, where 100 clonal switchgrass “Alamo” plants transgenic for an orange fluorescent protein (OFP) were used as the pollen source (whole plants, including pollen, were orange fluorescent). To assess pollen movement, pollen traps were placed at 10-m intervals from the pollen-source plot in the four cardinal directions extending up to 100 m from the field. Results showed that pollen-mediated gene flow is likely to occur over distances of at least 100 m (Millwood et al., 2017). This study provided important baseline data useful to determine isolation distances and other management practices, should transgenic switchgrass be grown commercially in relevant environments. Since switchgrass is an obligate outcrossing perennial grass, there are concerns about gene flow and the need for bio-confinement, especially for pollen (Kausch et al., 2010; Kwit & Stewart, 2012; Stockdale & Millwood, 2023). Moreover, since North America is the geographic center of switchgrass diversity, a better understanding of pollen movement in this species is needed (Kwit & Stewart, 2012).

The spread of pollen through the atmosphere involves processes of liberation, drift, and deposition (Aylor et al., 2003; Isard & Gage, 2001). Knowledge of these processes can help growers and producers make informed management decisions regarding pollen transport in seed production fields and neighboring farms (Isard & Gage, 2001)—and can inform allergen monitoring efforts in surrounding communities. Although atmospheric transport models can predict pollen movement, they often fail to incorporate actual measurements of pollen concentrations and viability. Various unmanned aircraft systems (UASs or drones) have previously been used to detect and monitor pollen movement over long distances in the lower atmosphere. Gottwald and Tedders pioneered the collection of pollen with UASs (Gottwald & Tedders, 1985). They modified a remote-controlled biplane platform with two rotating drum samplers to collect pollen and plant pathogen spores over pecan and peach orchards. Their study demonstrated the significant potential for regional-scale transport of pollen and plant pathogens among orchards. Two decades later, Aylor and colleagues (Aylor et al., 2006) combined ground-based sampling devices with UASs to collect pollen within and above a cornfield. Over the past decade, Schmale and colleagues have integrated autonomous systems into UASs, enabling teams of vehicles to coordinate flight missions and perform complex atmospheric sampling tasks (Schmale et al., 2008; Techy et al., 2010).

Improved pollen measurement also has direct public health implications. The allergen-management community needs a fast and reliable sensor network to measure airborne pollen concentrations to enable timely and accurate allergen reporting (Beggs, 2024; Buters et al., 2024; Suanno et al., 2021; Tummon et al., 2021). Current allergen information reports broad species group concentrations, typically at a daily resolution at best (Buters et al., 2018; Tummon et al., 2021). Future airborne pollen forecasts could be enhanced by integrating known pollen emissions with large-scale atmospheric models. Understanding diurnal pollen release patterns could aid in allergen treatment and improve emission source data for potential forecast models (Buters et al., 2018). To our knowledge, most airborne pollen field studies and corresponding allergen reports rely on Hirst-type samplers (Plaza et al., 2022). These sampling devices are constrained by a relatively low sampling rate of approximately 10 L/min (Adamov et al., 2024; Plaza et al., 2022), necessitating either high airborne pollen concentrations or extended sampling durations to accurately characterize pollen levels.

We hypothesized that (1) wind-dispersed pollen from switchgrass could be tracked and quantified using orange fluorescent protein (OFP) expression, (2) a Lagrangian stochastic (LS) dispersal model could estimate pollen source strength in the field, and (3) an array of novel samplers could serve as viable alternatives to standard Hirst-type samplers. To test these hypotheses, we conducted a series of unique release-recapture field studies using GE switchgrass in Oliver Springs, TN, USA. Two hundred plants from five transgenic lines of switchgrass (*Panicum virgatum* L. “Performer”) were planted at the center of a clear-cut field. One block consisted of 100 plants expressing OFP under the control of a switchgrass ubiquitin promoter (PvUBI1), while the other block contained 100 plants expressing OFP driven by a maize pollen-specific promoter (Zm13). Pollen from the atmosphere surrounding these blocks of transgenic switchgrass was collected using a series of fixed (ground-based) and mobile (drone-based) sampling devices at various distances from the field center. The efficacy of these various samplers was evaluated within 25 m of the source and up to 1 km from the source. LS dispersal simulations were conducted for pollen sampling intervals using high-resolution wind measurements collected near the field. Pollen emission rates were estimated by combining simulated concentrations with field concentration measurements. By integrating high-resolution measurements and simulations, our study evaluates the performance of emerging sampling technologies and highlights their implications for biosecurity, allergen tracking, and ecological modeling.

## Materials and methods

Three field campaigns were conducted over the course of two calendar years (2021 and 2022) to sample airborne pollen around two blocks of transgenic switchgrass.

**Fig. 1:**
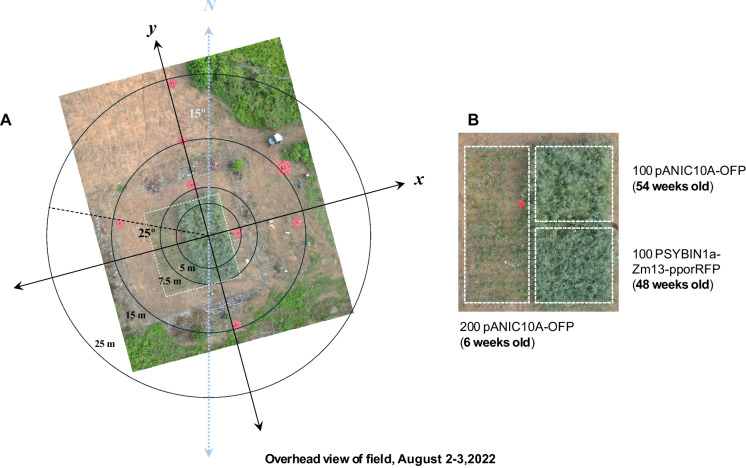
Top-down drone image of the field during field experiments conducted on August 2–3, 2022. **A** The field of GE switchgrass is outlined by a white dotted square and enclosed by a perimeter fence, as required by the APHIS BRS permits. Reddish-orange circular pads mark the locations of pollen sampling devices, positioned at increasing distances from the center of the source field. **B** A close-up view of the 14.5 m × 6.9 m field of GE Switchgrass, showing the locations of both strains of OFP-expressing switchgrass plants. Labels indicate the plant positions and ages during the August 2–3, 2022, field campaign

### Field site and pollen source

#### Field site

To assess the transport of wind-dispersed pollen from transgenic switchgrass plants, a 2-year field study was conducted under USDA APHIS BRS release permits (21-094-103r and 124-86SS5F1). The experiments were carried out at the Tennessee Agricultural Experiment Station, near the University of Tennessee’s Forest Resources Research and Education Center at the Cumberland Forest Unit in Oliver Springs, TN, USA (36.0483147, −84.4811417).

The field site was selected to satisfy the primary requirements for regulatory transgenic pollen dispersal experiments: isolation and traceable source attribution. It was situated on a patch of recently cleared forest land, with felled trees forming a rough glade area. The site provided sufficient open area (∼1.5 ha) for switchgrass cultivation and sampler deployment, while heavily forested borders served as a natural barrier that reduced the likelihood of cross-contamination with nearby wild or cultivated switchgrass and limited off-site pollen transport. The surrounding forest also sheltered the site from strong winds. The field location was intentionally chosen in a remote, concealed area beyond a secured access point, ensuring restricted visibility and access. The field plot measured 14.5 m × 6.9 m, enclosed within a protective 15.2 m × 19.8 m fenced perimeter, as shown in Fig. 1A. The outer fence was locked to prevent animal intrusion.

#### Transgenic line generation, analysis, and selection

Transgenic switchgrass plants expressing OFP were created by genetically engineering embryogenic callus derived from switchgrass seeds obtained from Ernst Conservation Seeds, Inc. (Meadville, PA, USA). This was achieved through Agrobacterium-mediated transformation (*Agrobacterium tumefaciens* strain EHA105) as detailed in Li and Qu (2011), using one of two binary plasmid constructs.

**Fig. 2:**
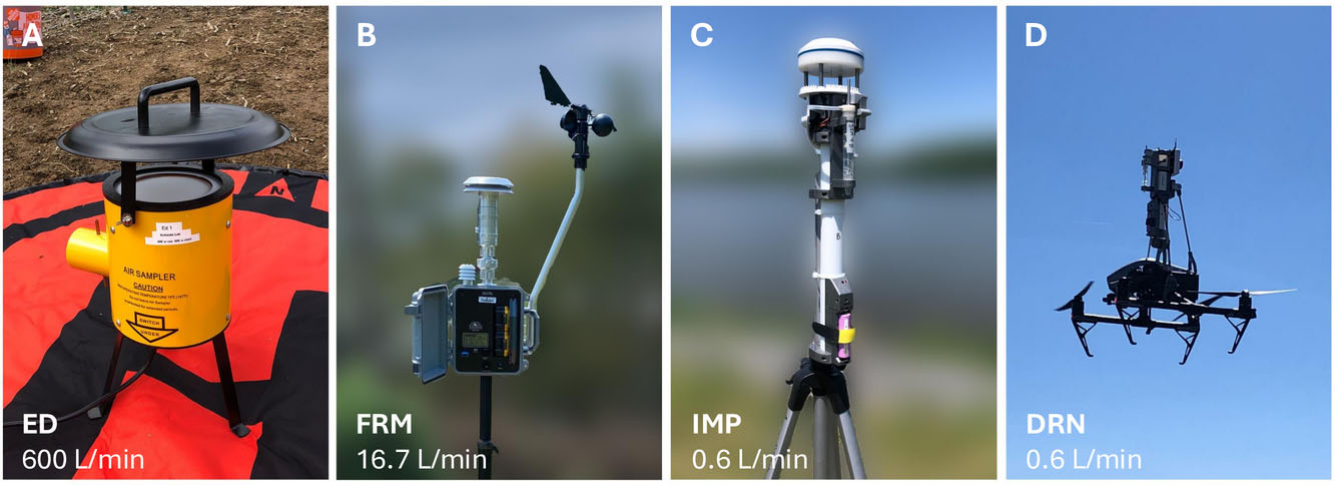
All sampler units used during the field campaigns. **A** The ED (Science First, 2007), a ground-based high-volume sampler (600 L/min). **B** The FRM, a ground-based medium-volume volumetric sampler (16.7 L/min). **C** The IMP, an impinger-based ground-based low-volume sampler (0.6 L/min). **D** The DRN, a drone-based sampler flown at a height of 10 m above ground level (0.6 L/min)

The first plasmid, pANIC10A-OFP (Mann et al., 2012), contained the hygromycin phosphotransferase (hph) selectable marker gene under the control of the switchgrass ubiquitin 2 (PvUbi2) promoter as well as an orange fluorescent protein (OFP) gene pporRFP under the control of the switchgrass ubiquitin promoter (PvUBI1). This promoter drives the expression of OFP in leaves, stems, and pollen.

The second plasmid, PSYBIN1a-Zm13-pporRFP, also contained the OFP gene pporRFP under the control of a maize pollen-specific promoter (Zm13). This promoter drives the expression of OFP in the pollen. This construct also contained a second OFP gene mOrangeER under the control of the cauliflower mosaic virus (CaMV) 35S promoter which enables the expression of this OFP in callus and green tissues. In addition, the plasmid also contained the hygromycin phosphotransferase (hph) selectable marker gene under the control of the PvUBI2 promoter. Several transgenic OFP-expressing shoots were recovered from hygromycin selection media (100 mg/L), and once rooted, plants were grown in an environmental-controlled growth room (16/8 h day/night and 24/22 °C day/night).

To confirm the presence of the OFP gene in the transgenic plants, PCR screening was performed using primers (forward primer, GCAAAGTGGGGTCAAAGATG; reverse primer, CACCTTCAAGCCCTTCTTTG) designed to amplify a 556 bp fragment of the pporRFP gene. PCR-confirmed transgenic plants were moved to a greenhouse and grown (16/8 h day/night and 28/22 °C day/night) until flowering. To identify transgenic events expressing OFP in pollen, visual analysis of OFP fluorescence was conducted on pollen grains from each event using epifluorescent microscopy as described by Rice et al. (2013). Transgenic lines in which all pollen grains exhibited OFP expression were propagated in the greenhouse and subsequently used in field experiments.

#### Planting

The planted area, less than 0.1 ha, consisted of 20 rows with 20 switchgrass plants per row, totaling 400 transgenic switchgrass plants arranged in a randomized design. These plants were hand-transplanted in the field at 76.2 cm intervals on three different dates. On July 20, 2021, 100 pANIC10A-OFP switchgrass plants from five transgenic events (20 clonal replicates per event) were transplanted. On August 26, 2021, another 100 PSYBIN1a-Zm13-pporRFP plants from five transgenic events (20 clonal replicates per event) were transplanted. Lastly, on June 20, 2022, an additional 200 pANIC10A-OFP switchgrass plants from ten transgenic events (20 clonal replicates per event) were transplanted in the field plot. These last 200 plants served as a contingency should earlier plantings fail. By the time of the field experiments, however, these were not mature enough to produce pollen and are not included in the analysis. Figure 1B illustrates the locations of these plants and their ages in weeks during the August 2–3, 2022, field experiment. This experimental design was structured to monitor and analyze the dispersal of transgenic pollen over time and distance.

### Sampling methods

**Fig. 3:**
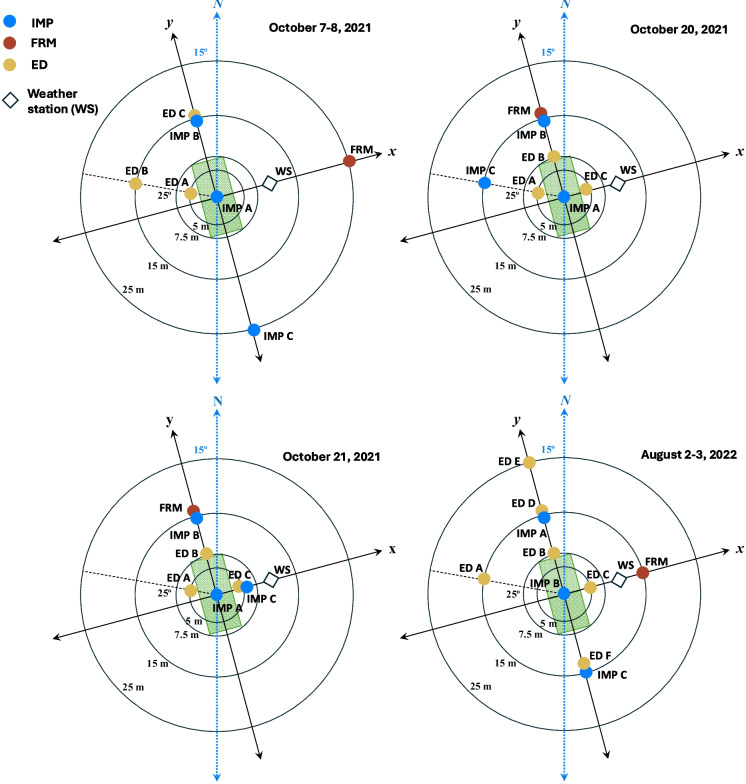
Ground-based sampler locations for each collection day. Yellow, blue, and red denote placement of ED, IMP, and FRM samplers, respectively, positioned at radial distances of 5, 7.5, 15, and 25 m from the center of the pollen-producing field (green rectangle). The diamond shows the location of the weather station, positioned 10 m East of the field

Four types of volumetric particle samplers (ED, FRM, IMP, and DRN), described in the following sections and shown in Fig. 2, were used to capture GE switchgrass pollen and estimate the concentration of airborne pollen at various times and distances (5, 7.5, 15, and 25 m) from the source field. Each sampler had different sampling rates and sensing capabilities, described in the sections below. Figure 3 illustrates the placement of samplers around the field on each sampling day. The samplers were placed on reddish-orange circular drone landing pads to mark their locations and enhance visibility in overhead drone footage, as shown in Fig. 1A for the August 2–3, 2022, field campaign.

#### Ground-based high-volume samplers (ED)

In anticipation of low pollen emission rates, several high-volume filter-based samplers (Science First #15000, Yulee, FL) (Science First, 2007) were deployed during the campaign (Fig. 2A). Originally designed for educational use in schools, these samplers are referred to as “ED” samplers throughout the manuscript.

The barrel-shaped ED samplers draw air through a filter surface at an initial flow rate of 600 L/min (Science First, 2007). Cellulose filters with a pore size of 11 μm and a diameter of 125 mm were used to collect airborne pollen and other atmospheric particles at 0.432 m above ground level. The ED samplers’ volumetric sampling rate is 1000 times that of the IMP and DRN samplers, and 35 times that of the FRM sampler. This significant increase in sampling capacity allowed for improved detection of airborne pollen, particularly in cases of low pollen emission rates.

#### Ground-based medium-volume sampler (FRM)

A single near-federal reference method (FRM) sampler (ARA Instruments, Eugene, Oregon) was deployed during the field experiments, shown in Fig. 2B. This battery-operated device samples air at a flow rate of 16.7 L/min. The unit is equipped with a filter sampler (PM$_{10}$ filters were used in this study), meteorological sensors, and a particle counter. Additional details about this instrument are available on ARA’s website (ARA Instruments, 2016).

#### Ground-based low-volume samplers (impingers or IMP)

To evaluate the effectiveness of impinger-type samplers, three custom-designed impinger packages were deployed during the field campaign (Fig. 2C). These sampling packages are referred to as “IMP” throughout the manuscript.

The IMPs were constructed from high-density polyethylene, following the design specifications outlined in Powers et al. (2018). The 3D printing (.stl) files for the impinger units are publicly available online (Powers, 2018). These files were modified to accommodate a 15-mL polypropylene conical collection vial (Corning #CLS430791) and a stainless-steel tube with a 4-mm diameter opening. The 4-mm inlet opening was substantially larger than switchgrass pollen grains (≈ 35 μm diameter) and therefore was not expected to impede pollen entry into the sampler. The collection pathway was also designed to minimize particle adhesion losses prior to deposition into the collection liquid, consistent with the validated impinger architecture described in Powers et al. (2018).

The IMP samplers were mounted on a tripod 2 m above ground level to approximate the height of the switchgrass panicles, the open flower structures that produce pollen. The IMPs sampled airborne particles at a rate of 0.6 L/min, with collected particles entrained in sterile 15-mL conical tubes containing 2 mL of sterile deionized water.

#### Drone-based low-volume sampler (DRN)

To measure airborne pollen concentration at different altitudes above and downwind of the source field, we used a drone-based sampling system consisting of the IMP unit mounted on a DJI Inspire 2 drone platform (Fig. 2D). The system is described in detail in Bilyeu et al. (2022).

A key design feature of the drone system is the positioning of the IMP sampler high enough above the propellers, which ensures that the sampled air remains free from propeller-induced turbulence, commonly known as downwash. The drone was flown at a fixed altitude of 10 m during each sampling interval, a height selected to prevent propeller downwash from disturbing the switchgrass canopy during stable hovering.

Due to drone battery limitations and the need for safe flight and landing operations, each sampling interval was restricted to 10 min. The IMP unit on the drone operated at the same volumetric sampling rate as its ground-based counterpart (0.6 L/min). However, because the drone sampler was only flown for 10 min per flight, its total sampling capacity was significantly lower than the ground-based IMP units, which collected for 30 to 90 min during the field campaign. Hereafter, the IMP-equipped drone system is referred to as “DRN” throughout the manuscript.

#### Sampler deployment during campaigns

The first field campaign took place on October 7–8, 2021. The following ground-based samplers were placed radially around the field at distances of 0, 5, 15, and 25 m, as shown in Fig. 3: three ED samplers, three IMPs, and one FRM sampler. The ground-based samplers operated in 30-min sampling intervals, while the drone-based sampler (DRN) was flown at a 10-m height for a duration of 10 min per sampling interval. Due to technical difficulties, the drone sampler was only deployed on October 7th and was not used on October 8th. IMP A was placed at the center of the field (the midpoint of fields 1 and 2) with the intention of estimating pollen emission rate. IMP B and ED C were co-located with the intention of comparing concentration estimates between samplers.

During the second field campaign on October 20–21, 2021, sampling intervals for all ground-based samplers were increased from 30 to 90 min to improve pollen counts. For the same reason, all samplers were moved closer to the field, within 15 m of the field. The drone sampling interval was 10 min. On October 21st, the final sampling interval was halted early due to an incoming storm.

The third and final campaign took place on August 2–3, 2022. Field and sampler placements during this campaign are shown in Figs. 1A and 3. Given that ED samplers were the most effective in previous campaigns, their number was increased from three to six. In anticipation of prevailing winds directed toward north-northeast, samplers were primarily aligned along the positive *x* and *y* axes in Figs. 1 and 3. The drone sampler was again flown for 10 min at 10 m during all sampling intervals. IMP B was placed at the field center with the intention of estimating pollen emission rate. IMP A and ED D were co-located with the intention of comparing concentration estimates between samplers. To address diurnal trends, sampling intervals were kept consistent across both collection days. All ground-based samplers were operated for 45-min sampling intervals.

### Processing of pollen samples

#### Sample preparation

**Fig. 4:**
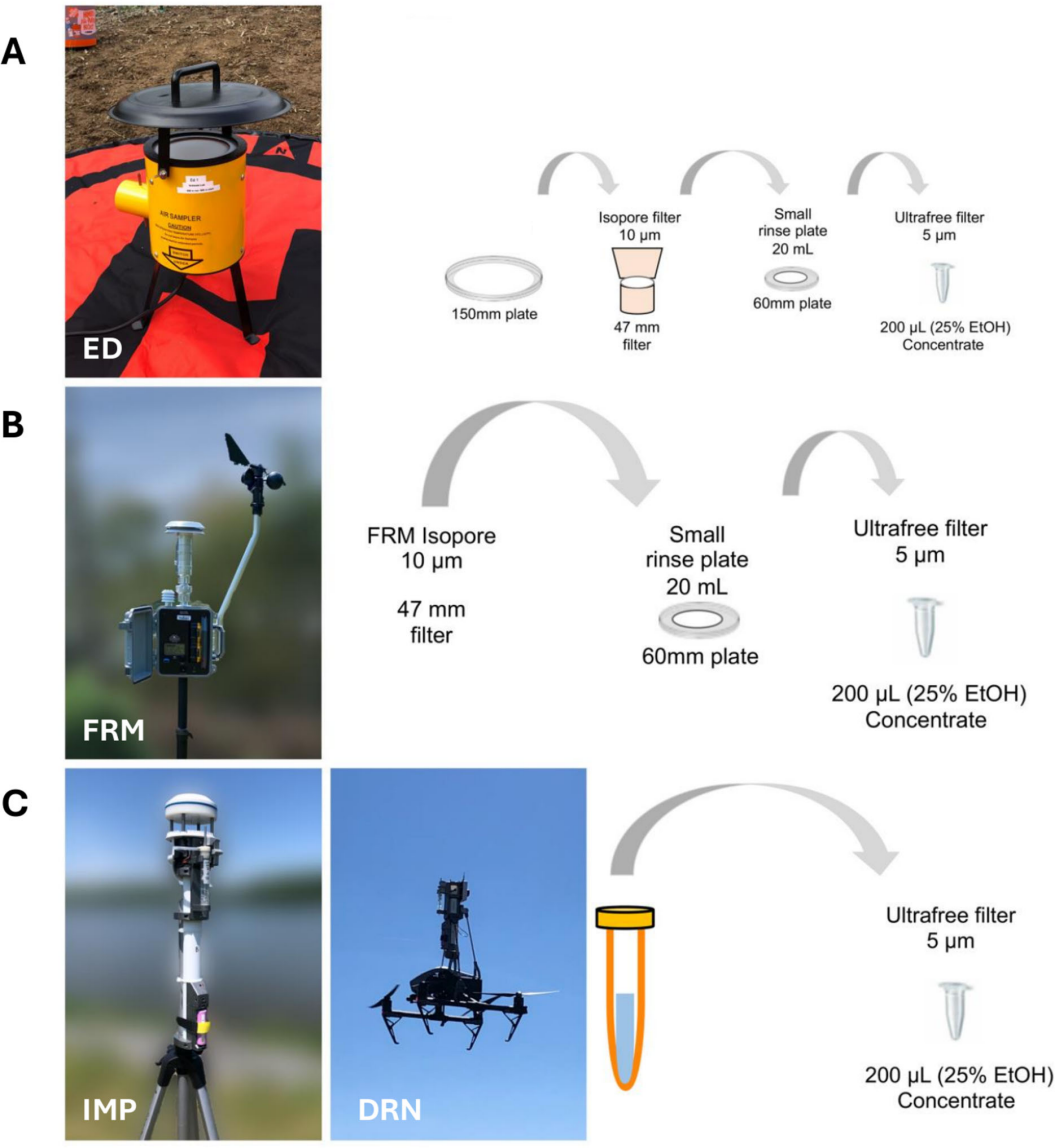
Flow charts showing the processing of the filters from **A** the ED samplers, **B** the FRM sampler, and **C** the IMP and DRN samplers

Filters from the ED samplers were processed as shown in Fig. 4A. Briefly, the 125-mm “ED” collection filters were removed with forceps and transferred to separate 150-mm petri dishes (Fisher #FB0875714) in the field immediately following each sampling interval. For each filter, 25 mL of 25% EtOH was added to the petri dish, the filter was gently agitated with a sterile cell spreader, and then rinsed a total of eight times. Each rinsate was transferred by a pipettor to a vacuum filtration unit (Thomas Scientific #300-4100) containing a 47-mm Isopore polycarbonate 10 μm filter (Millipore Sigma #TCTP04700). The sample was cleared through the filter using the vacuum from a hand pipetting bulb. Using forceps, the Isopore filter was then moved to a 60-mm petri dish (Genesee 32-105) and rinsed six times with 2 mL 25% EtOH. The resulting rinsate was transferred to an Ultrafree 5 μm PVDF centrifugal filtration tube (Millipore Sigma UFC40SV25) and centrifuged for 2 min at 2500 rpm in a swinging bucket centrifuge (IEC Clinical Centrifuge). The concentrated sample was then resuspended from the 5-μm filter surface with 200 μL 25% EtOH and moved to a 1.5-mL Eppendorf tube and stored at 4 °C for further analysis.

Filters from the FRM sampler were processed as shown in Fig. 4B. Briefly, the Isopore filter was removed from the FRM unit sampling cartridge using forceps and transferred to a 60-mm petri dish (Genesee 32-105) in the field immediately following each sampling interval. For processing the sample, the Isopore filter was then rinsed six times with 2 mL 25% EtOH, and the resulting rinsate was moved to an Ultrafree 5 μm PVDF centrifugal filtration tube and centrifuged for 2 min at 2500 rpm in a swinging bucket centrifuge (IEC Clinical Centrifuge). The concentrated sample was then resuspended from the 5-μm filter surface with 200 μL of 25% EtOH and moved to a 1.5-mL Eppendorf tube and stored at 4 °C for further analysis.

The fluid from the IMP and DRN samplers was processed as shown in Fig. 4C. Samples from the IMPs and DRN were transferred by pipette to an Ultrafree 5-μm PVDF centrifugal filtration tube and centrifuged for 2 min at 2500 rpm in a swinging bucket centrifuge (IEC Clinical Centrifuge). The concentrated sample was then resuspended from the 5-μm filter surface with 200 μL 25% EtOH and transferred to a 1.5-mL Eppendorf tube and stored at 4 °C for further analysis.

#### Pollen counting

Switchgrass pollen was counted in each concentrated sample by pipetting the samples into individual wells of a 96-well plate (Grenier Bio One 7000124). Samples were allowed to sediment for 15 min and then were observed using an Olympus CKX53 inverted microscope equipped with the Olympus EP50 digital camera and associated software. Following the quantification of the switchgrass pollen in each of the tubes, the samples were transferred back into their respective 1.5-mL Eppendorf storage tubes and held at 4 °C for transport and further analysis.

### Meteorological data

A weather station was installed near the field to collect meteorological data throughout each sampling day. The station consisted of a Campbell Scientific CSAT3 three-dimensional sonic anemometer, mounted at a height of 1.5 m above ground level, which measured high-resolution wind velocity in three dimensions and sonic temperature at a frequency of 10 Hz. In addition, a Campbell Scientific HMP45C probe recorded temperature and relative humidity every 30 s. Meteorological data were recorded with the Campbell Scientific CR3000 datalogger. To minimize interference from the tripod pole, the sonic anemometer arm was positioned perpendicular to the anticipated dominant wind direction before each collection day. The wind velocities in the *u* and *v* directions (relative to the sonic anemometer arm) were then rotated into the cardinal coordinate system for analysis.

### Atmospheric dispersal modeling

#### Meteorological Inputs

Atmospheric dispersal simulations were driven using time-averaged wind statistics from the weather station (“Meteorological data”) collected during each sampling interval. Most sampling intervals occurred under low-wind conditions (< 2 m/s), likely due to the sheltered field site, characterized by meandering winds with frequent directional shifts and intermittent lulls in wind speed. To better capture dispersal dynamics under these conditions, the wind data were processed using different averaging window sizes. Specifically, for the 45-min sampling intervals on August 2–3, 2022, the following averaging windows were used: 45 1-min averaging windows, 9 5-min averaging windows, and 1 full 45-min averaging window. This approach allowed for assessing how different temporal resolutions of wind averaging influenced the accuracy of dispersal simulations.

To compute turbulence statistics for each averaging window, the average downwind direction was determined, and the wind velocity data were rotated into a downwind (*u*) and crosswind (*v*) coordinate system. For each sampling interval, the means, covariances, and variances were computed for these wind velocity components, as well as temperature. Mean temperature was computed from sonic temperature using the method described in Schotanus et al. (1983) and the relative humidity values. Heat flux was estimated from sonic temperature and relative humidity using the Bowen ratio method from Schotanus et al. (1983). The Bowen ratio was determined using the simplified method of Lin et al. (2016), which requires only mean temperature and relative humidity. These turbulence statistics provided the necessary meteorological inputs for the dispersal simulation in each interval, specifically friction velocity ($u_*$) and the Monin-Obukhov length (*L*).

#### Pollen dispersal simulations

Switchgrass pollen dispersal was simulated using a custom MATLAB implementation of the surface-layer Lagrangian stochastic (LS) model described in Aylor and Flesch (2001) and expanded to three-dimensional transport in Aylor (2017). The LS model framework is based on Brownian motion theory, modeling turbulent diffusion by simulating the trajectories of thousands of particles through the air as random walks through the atmosphere. The movement of each particle is governed by turbulent wind statistics, and the ensemble average of these trajectories provides estimates of pollen concentration at any given location within the simulation domain.

LS models require turbulent wind statistics to be specified at every point in the simulation domain, including mean velocities, variances, and covariances of the wind components. Under the assumptions of stationarity and horizontal homogeneity, these wind field statistics remain constant over time within each averaging window but vary with height. To account for this height dependence, boundary layer scaling techniques are applied to generate vertical wind profiles based on measurable surface-level parameters, specifically the friction velocity ($u_*$) and the Monin-Obukhov length (*L*). The full model formulation and wind statistics used in the simulations are included in the code provided online.

These two parameters ($u_*$ and *L*) were computed from the time-averaged meteorological measurements for each 45-min sampling interval, using three different averaging approaches: one 45-min averaging window; nine 5-min averaging windows; and 45 1-min averaging windows. A separate simulation was conducted for each averaging window in a 45-min sampling interval, using the computed average wind direction, friction velocity ($u_*$), and Monin-Obukhov length (*L*). The resulting concentration fields from these simulations were then averaged to generate a single mean concentration field for each 45-min sampling interval.

In each simulation, 100,000 particles (representing switchgrass pollen) were released from a point source at the center of the field at a height of 2 m, which approximates the height of most of the switchgrass panicles in the field experiment. A point-source approach was chosen following established methodology (Aylor & Flesch, 2001) and for computational simplicity. A spatially distributed source could better represent the field geometry, but would require assuming uniform emission across the planted area, which is unlikely given that individual plants vary in maturity, wind exposure, and panicle development. Particles were removed from the simulation domain when they: (1) traveled more than 50 m laterally from the source, (2) rose above 100 m above ground level, or (3) fell below 0.1 m above ground level. To simplify the simulation, pollen dispersion was modeled as if it occurred over a flat, rough surface, with an estimated surface roughness of 0.01 m, consistent with values reported for level grassy plains and prairie in Hansen (1993). The settling velocity was estimated as 0.0371 m/s, based on an observed switchgrass pollen diameter of ∼35 μm, using Stokes’ law. Since switchgrass pollen was observed to be nearly spherical, Stokes’ law provides a reliable approximation of its settling velocity.

#### Concentration estimation and source emission rate calculation

The pollen concentration estimation procedure in this study follows the approach described by Flesch et al. (1995) for a stationary LS model with a constant source. Pollen concentration is estimated by tracking the amount of time particles spend in each grid box, normalized by the total number of particles released ($N_\textrm{p}$) and the volume of the grid box ($V_\textrm{box}$ = 1 m × 1 m × 1 m), and then multiplied by the modeled emission rate (*Q*). Specifically, the concentration at a given grid point (*i*, *j*, *k*) is calculated as

1 $$\begin{aligned} C(i,j,k)&= Q \frac{1}{V_\textrm{box}}\frac{1}{N_\textrm{p}}\sum _{n=1}^{N_\textrm{p}}T_n(i,j,k), \end{aligned}$$

where $T_n(i,j,k)$ represents the time particle *n* spends in the given grid box. Ground-level concentrations reported throughout this study correspond to the first vertical grid cell (0–1 m above ground).

We employed a model-measurement fusion approach described in Aylor and Flesch (2001) to estimate pollen emission rate and concentration. For each sampling interval, the modeled pollen concentration at each grid point in the simulation domain is first computed under the arbitrary assumption of a constant release rate at the center of the field of $Q_\textrm{model}$ = 1 particle per second. This yields a modeled relative concentration, which is proportional to the actual concentration at every point in the domain. To estimate the actual emission rate (pollen flux from the field), the ratio of the measured pollen concentration at each of the six ED samplers to the modeled relative concentration at the corresponding locations in the simulation domain was computed and used to update the value of *Q*. To obtain a single emission rate estimate for each sampling interval, the computed emission rates corresponding to calculations based on each of the six ED samplers were averaged. This estimated true emission rate was then used to update the modeled relative concentration to predict the actual concentration at every point within the simulation domain.

To investigate dispersal at greater distances—up to 1 km from the source—the same modeling procedure was conducted but with a coarser grid resolution of $V_\textrm{box}$ = 3 m × 3 m × 1 m and an extended simulation domain covering 1000 m × 1000 m × 100 m. This coarser grid was selected to balance computational efficiency while maintaining consistency with the finer-resolution near-source grid.

To generate 2D dispersal kernels, which represent pollen concentration as a function of distance, concentrations at equal radial distances from the source are averaged, yielding an average concentration as a function of radial distance from the source.

**Table 1:** Pollen concentrations (pollen/m$^3$) sampled during specific time periods, organized by date and sampler type

		ED						FRM	IMP			DRN^**^
Date	Time	A	B	C	D	E	F		A	B	C	
7 Oct 2021	11:30–12:00	0	0.17	0	-	-	-	0	0	-	0	0
	12:35–13:05	0	0	0.06^†^	-	-	-	0	0	0	0	0
	13:45–14:15	0.06^†^	0	0	-	-	-	0	0	0	0	0
	14:45–15:15	0.11^†^	0	0.11^†^	-	-	-	2.00^†^	0	0	0	0
	15:50–16:20	0.17	0.06^†^	0.11^†^	-	-	-	0	0	0	0	0
8 Oct 2021	12:00–12:30	0	0	0.33	-	-	-	2.00^†^	0	0	0	-
	13:00–13:30	0.17	0.17	0.06^†^	-	-	-	0	0	0	0	-
	14:00–14:30	0	0	0.22	-	-	-	0	111.11^†^	0	0	-
	15:00–15:30	0.11^†^	0.17	0.33	-	-	-	0	0	0	0	-
20 Oct 2021	14:00–16:00^*^	0.02^†^	0.02^†^	0.02^†^	-	-	-	0.67^†^	0	0	0	0
	16:30–18:15^*^	0.02^†^	0.02^†^	0.02^†^	-	-	-	0	0	0	0	0
21 Oct 2021	12:00–13:45^*^	0	0	0.02^†^	-	-	-	0	0	0	0	0
	14:00–14:50	0	0	0.03^†^	-	-	-	0	0	0	0	0
2 Aug 2022	10:00–10:45	0.37	0.48	2.48	0.15	0.04^†^	0.04^†^	0	0	0	0	0
	11:00-11:45	0.11	0.89	0.59	0.04^†^	0.04^†^	0.04^†^	0	0	0	0	0
	12:00–12:45	0.07^†^	3.19	1.04	0.37	0.22	0.11	0	0	0	0	0
	13:00–13:45	0.22	4.30	31.78	0.33	0.26	1.89	0	0	0	0	-
	14:00–14:45	0.37	41.44	19.74	4.67	1.00	1.59	15.97	74.07^†^	37.04^†^	0	166.67^†^
	15:00–15:45	0.26	5.41	5.85	0.15	0.11	0.22	3.99	0	0	37.04^†^	0
3 Aug 2022	10:00–10:45	0.04^†^	0.15	0.33	0	-	0.07^†^	0	0	37.04^†^	0	333.33^†^
	11:00–11:45	0	1.74	0.30	0.07^†^	0	0.15	0	0	0	0	0
	12:00–12:45	0	0.48	0.85	0.04^†^	0.15	0.07^†^	0	0	0	0	0
	13:00–13:45	0.15	6.22	17.89	0.74	0.04^†^	0.11	1.33^†^	0	37.04^†^	37.04^†^	0
	14:00–14:45	15.11	23.44	29.26	9.67	1.70	3.19	10.65	0	444.44	74.07^†^	500.00
	15:00–15:45	0.78	5.19	9.07	1.44	-	0.48	13.31	111.11	185.19	37.04^†^	0

^†^Only 1–2 pollen grains were sampled. ^*^For these sampling intervals, sampling times for ED and FRM were limited to two 45-min intervals with a break in between to prevent overheating, resulting in 90 min of total sampling time. Impingers sampled for the entire time. ^**^Drone was sampling for a total of 10 min in each sampling interval due to battery limitations

## Results

### Field experiments

The resulting measured pollen concentrations for each field campaign, sampling interval, and sampler are presented in Table 1. Concentrations marked with a † denote cases where only 1–2 pollen grains were sampled—insufficient for meaningful statistical analysis. For example, the high concentrations often reported by the IMP and DRN samplers (e.g., 111.11, 166.67, or 333.33 pollen/m$^3$) are likely artifacts of detecting few grains in very small sample volumes rather than genuinely high airborne concentrations.

#### First campaign (October 7–8, 2021)

The first field campaign took place on October 7–8, 2021, when the PSYBIN1a and pANIC10A plants were only 6 and 11 weeks old, respectively, and the youngest 200 pANIC10A plants had not yet been planted. The immaturity of these plants resulted in negligible pollen production during this campaign.

As shown in Table 1, across both sampling days, no sampler captured more than five pollen grains in any single interval, and most readings were zero. On October 7th, maximum captures were three grains per sampler. October 8th showed marginally higher counts of 1–5 grains per sampler, with ED C performing slightly better than other samplers, particularly during afternoon sampling intervals.

The low pollen counts limited the feasibility of the intended analyses, but helped us prepare for future experiments. IMP A, positioned at the field center to estimate emission rates, collected at most two pollen grains per interval—far below the threshold needed for emission rate calculations. Similarly, the co-located IMP B and ED C could not provide meaningful inter-sampler comparisons due to their vastly different volumetric sampling rates (0.6 L/min vs 600 L/min) combined with negligible pollen counts. IMP B captured zero pollen across most intervals, while the few instances of apparent detection (e.g., 111.11 pollen/m$^3$ on October 8th) represent the limitations of low-volume samplers.

#### Second campaign (October 20–21, 2021)

The second field campaign took place on October 20–21, 2021. Sampling intervals were extended to 90 min, and all samplers were repositioned within 15 m of the field to improve pollen capture from the same plant populations.

Pollen counts remained low throughout the campaign (Table 1), with maximum captures of 1–2 grains per sampler. ED A, B, and C detected 1–2 grains per 90-min interval when pollen was present. The FRM sampler detected pollen in one interval (0.67 pollen/m$^3$). All IMP and drone samplers recorded zero. Heavy rainfall on October 21st required early termination of the final sampling interval. The extended sampling durations and closer placement were insufficient to enable the intended analyses.

#### Third campaign (August 2–3, 2022)

The third and final campaign took place on August 2–3, 2022, when the oldest pANIC10A plants were at peak pollen production. The PSYBIN1a plants had not yet begun releasing pollen, and the youngest pANIC10A field was still too immature to contribute. Six ED samplers were deployed instead of the previous three.

Pollen concentrations are presented in Table 1. ED samplers collected substantially more pollen than in previous campaigns, with concentrations ranging from near-zero during morning hours to over 40 pollen/m$^3$ during peak periods. ED B and C consistently showed the highest concentrations, reaching maximum values of 41.44 and 31.78 pollen/m$^3$, respectively, during the 14:00–14:45 intervals. A clear diurnal pattern emerged on both days, with concentrations beginning to increase around 13:00, peaking between 14:00 and 14:45, and then declining after 15:00.

The higher ED sampler concentrations provided a robust dataset for estimating the emission rate using atmospheric dispersal modeling. The IMP and FRM samplers remained limited by their lower sampling volumes, with most IMP readings still representing only 1–2 grains (marked †) despite the increased source strength. The co-located ED D and IMP A illustrated the sampling rate constraints, with ED D detecting meaningful concentrations while IMP A remained near detection limits.

**Fig. 5:**
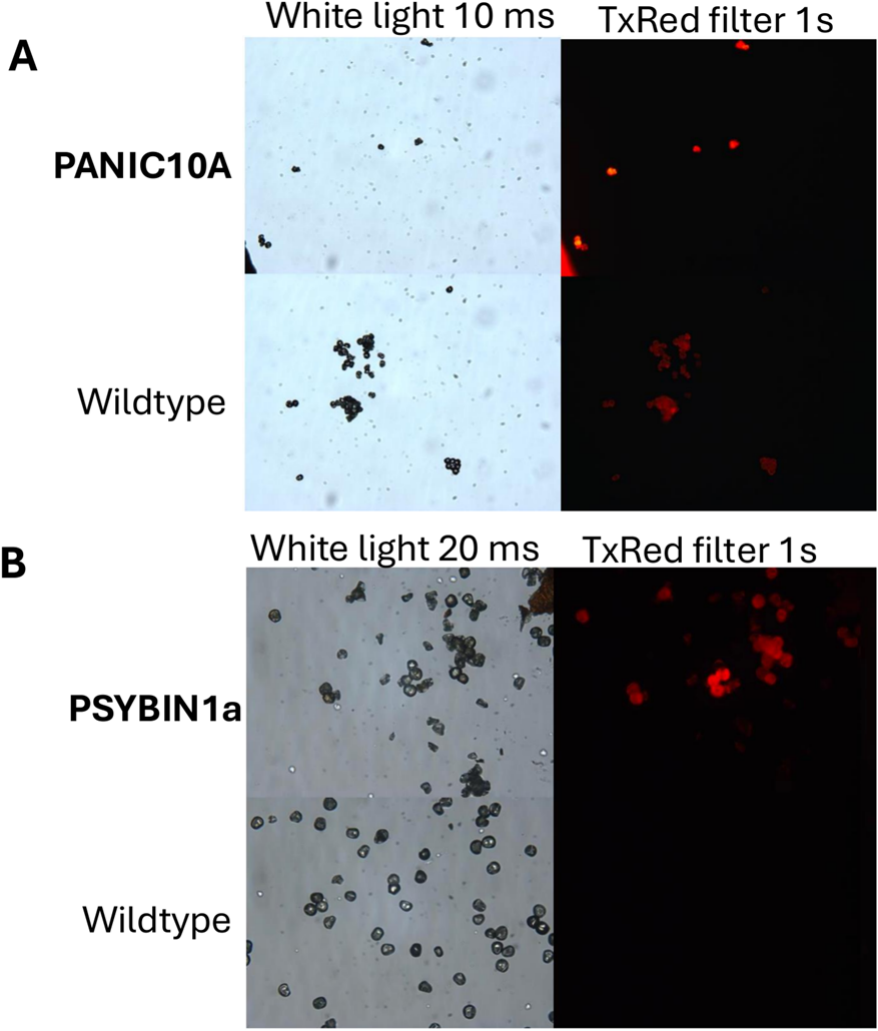
Comparison of OFP signal between GE switchgrass pollen and wild-type. **A** Pollen from the pANIC10A strain (OFP expression throughout the entire plant) is not easily distinguishable from wild-type pollen under OFP-inducing light. **B** Pollen from the PSYBIN1a strain (OFP expression restricted to pollen) exhibits a strong, highly distinguishable OFP signal compared to wild-type pollen

### Orange-fluorescent protein expression

The primary source of pollen in the field experiments came from the first planted block of GE switchgrass, which contained 100 plants expressing OFP under the control of a switchgrass ubiquitin promoter (PvUBI1). As shown in Fig. 5, the OFP signal in pollen grains from these transgenic plants was difficult to distinguish from wild-type (WT) pollen exposed to the same OFP-inducing wavelength of light. Some level of autofluorescence was expected in WT pollen, as pollen walls naturally contain autofluorescent compounds including sporopollenin, flavonoids, and other phenolic constituents (Donaldson, 2020). This endogenous fluorescence likely contributed to the background signal observed under OFP excitation conditions. In contrast, the OFP signal in pollen from the later planting of GE switchgrass (expressing OFP under a maize pollen-specific promoter, Zm13) was much stronger and easily distinguishable from WT pollen. However, these Zm13-expressing plants were smaller and did not produce sufficient mature panicles in time for the field experiments, limiting their contribution to the study.

### Modeling results

For the dispersal modeling, we focused on the third field campaign (August 2–3, 2022), as sufficient pollen was captured on both days to allow for concentration measurements from the ED samplers. All sampling intervals during these days were 45 min long. Sampling occurred at consistent times across both days, facilitating comparisons and enabling the identification of diurnal trends.

#### Near-source concentration and emission rate estimation

**Fig. 6:**
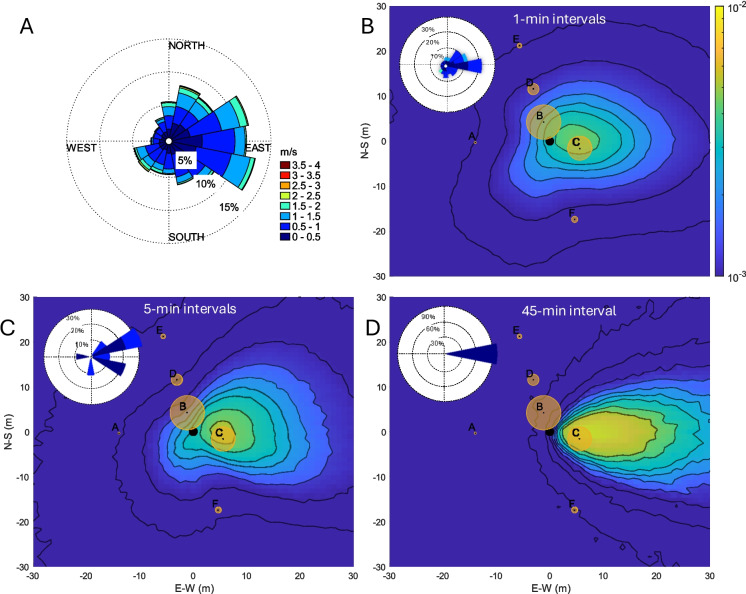
Relative ground level concentration contours for the August 2nd, 14:00–14:45 sampling interval. **A** Wind rose for this sampling interval, indicating the downwind direction. **B** Contour plot averaging 45 1-min simulations. **C** Contour plot averaging nine 5-min simulations. **D** Contour plot generated using a single 45-min simulation. Yellow circles indicate the ED sampler locations, with their size proportional to pollen counts at each site

Dispersal simulations more accurately capture changing wind directions and pollen concentrations when shorter averaging windows are used for each sampling interval. Figure 6 presents ground-level relative concentration contours and downwind wind roses for the August 2nd, 14:00–14:45 sampling interval, simulated using 1-min, 5-min, and 45-min averaging windows. This interval corresponded to the highest measured pollen concentrations by the ED samplers. The wind rose in Fig. 6A was generated using wind data collected at a sampling frequency of 10 Hz. The wind roses in Fig. 6B, C, and D were created using 1-min, 5-min, and 45-min time-averaged wind data, respectively. Wind roses display the downwind direction. Yellow circles indicate the ED sampler locations, with their size proportional to pollen counts at each site.

*Comparison of averaging windows*  The 45-min plot (Fig. 6D), based on a single LS simulation in the average downwind direction, fails to capture wind variability and lateral pollen spread, missing high pollen counts at ED sampler B due to a single eastward-directed plume. The 5-min plot (Fig. 6C), which averages nine LS simulations, shows some directional variation but lacks the detail seen in the 1-min plot. The 1-min plot (Fig. 6B) provides the most accurate representation of dispersal dynamics. However, all three simulations share a common discrepancy: peak concentrations appear a few meters from the point source, indicating lateral transport before deposition. These near-source discrepancies may reflect the point-source and flat-surface simplifications discussed in Section 2.5.3.

**Fig. 7:**
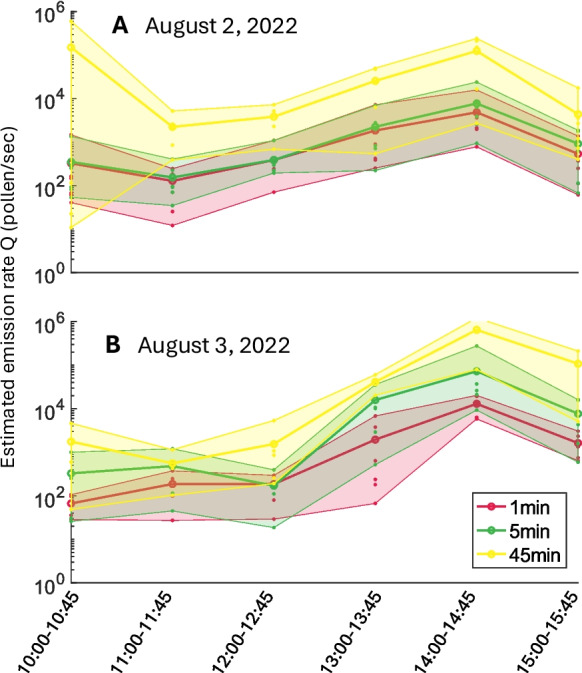
Mean estimated pollen emission rates for each sampling interval using 1-min, 5-min, and 45-min simulations. **A** Estimated emission rates for August 2, 2022, and **B** August 3, 2022. Non-zero emission rate estimates for each sampler are shown as solid points. Shaded regions indicate the range between the minimum and maximum non-zero emission rate estimates. The vertical axis is on a log scale

*Emission rate and diurnal pattern*  The computed pollen emission rate from the field exhibits a clear diurnal trend. Figure 7 presents the mean, minimum, and maximum non-zero computed emission rates for each sampling interval on August 2–3, 2022, during the third field campaign. Emission rates were computed from Eq. 1, based on the ratio of measured to modeled concentrations. Samplers with zero measured or modeled concentrations were excluded to prevent infinite or zero emission rate estimates. Emission rate calculations were performed for 1-min, 5-min, and 45-min averaging windows. The range of estimated emission rates decreases with smaller averaging windows. The pollen emission rate increased by approximately three orders of magnitude between 10:00 and 14:00. Note that the emission rate is shown on a log scale in Fig. 7. The log-transformed emission rate estimate is positively correlated with the horizontal velocity magnitude (Pearson’s *R* = 0.73, *P* = 0.01), temperature (*R* = 0.71, *P* = 0.01), and vapor pressure deficit (*R* = 0.69, *P* = 0.01), while negatively correlated with relative humidity (*R* = −0.63, *P* = 0.02). These results indicate that higher pollen emissions occur under conditions of higher wind speed, temperature, and vapor pressure deficit, while increased relative humidity reduces pollen release.

**Fig. 8:**
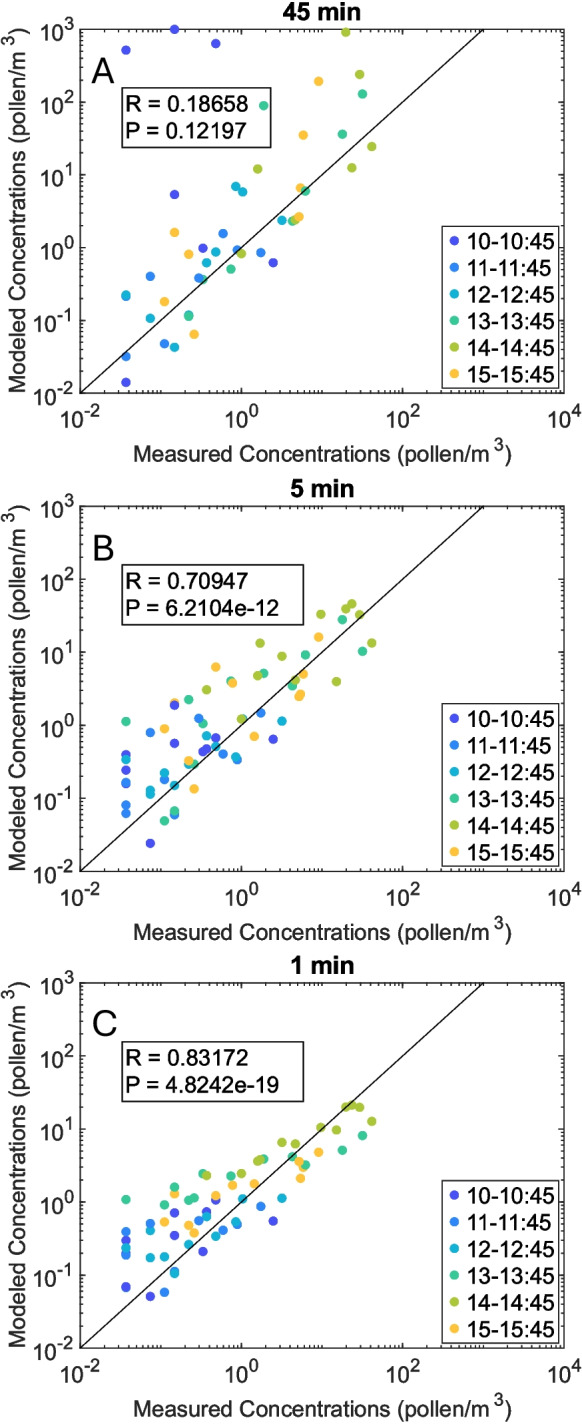
Measured concentrations at the ED samplers compared with simulated concentrations for **A** 45-min, **B** 5-min, and **C** 1-min intervals. These plots are not intended as model validation, but rather to show that decreasing the averaging time for simulations greatly improves modeled concentrations. Note that the plots are on log-log scales

Modeled concentration predictions improve dramatically when changing wind conditions are incorporated into the simulations. Figure 8 compares modeled concentrations, computed by multiplying the estimated emission rate by the relative concentration, with measured concentrations derived from ED sampler pollen counts. Although the figures directly compare modeled and measured concentrations, they are not intended as a formal model validation, as the measured concentrations were directly used to compute emission rate and modeled concentration (see Section 2.5.3). Instead, they highlight the substantial improvement in model performance as the averaging window is reduced. Pearson’s *R*-value increases from 0.19 for 45-min windows to 0.71 for 5-min windows to 0.84 for 1-min windows. While reducing the averaging window from 45 to 5 min requires nine times the computational power, it yields a 270% improvement in model performance (as measured by the *R*-value). In contrast, refining the resolution further from 5- to 1-min windows results in only a 20% increase, suggesting that shorter windows may not always be computationally worthwhile beyond a certain threshold.

#### Estimating sensor capabilities in the far field

Given that only the ED samplers collected sufficient pollen in this study, we estimated how each sampler type would perform under different source strengths and at greater distances from the field. The four sampler types differ in intake rate by multiple orders of magnitude: 0.6 L/min for the IMP and DRN, 16.7 L/min for the FRM, and 600 L/min for the ED. Because pollen grains arrive as discrete counts, lower intake rates require proportionally higher airborne concentrations to collect enough grains for a reliable measurement. The Poisson distribution models this counting error as $\sqrt{N}$, where *N* is the number of grains collected (Addison-Smith et al., 2020; Lin et al., 2013). We selected a threshold of 100 pollen grains, which ensures ±10% error in concentration estimates.

**Fig. 9:**
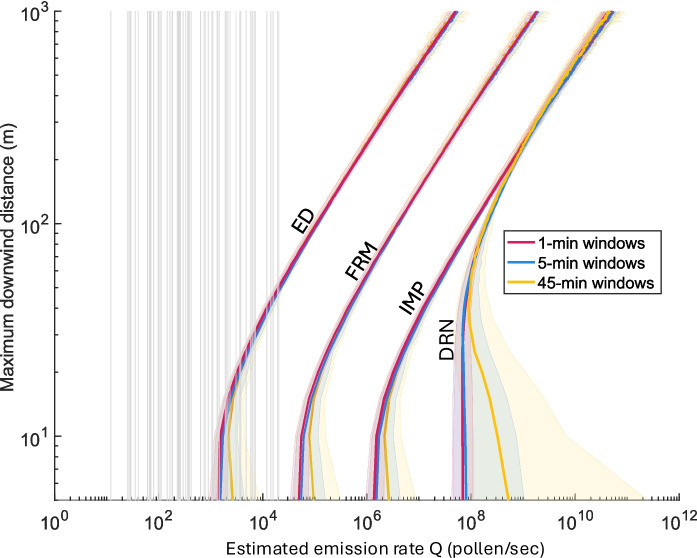
Maximum distance at which ED, FRM, IMP, and DRN sensors should be placed from the source to capture at least 100 pollen grains for a given emission rate from the field. These are calculated using concentration curves derived from each sampling interval, distinguishing between estimates computed with 1-min, 5-min, and 45-min averaging windows. Solid lines represent the median values, while the shaded regions indicate the range between the lowest and highest values observed across all sampling intervals. Gray vertical lines correspond to the estimated emission rates for each sensor during all sampling intervals

Using the long-distance 2D relative concentration dispersal kernels from our simulations, we computed the maximum distance from the source at which each sampler could collect at least 100 grains (±10) within a 45-min sampling interval, as a function of emission rate (Fig. 9). Ground-based samplers (ED, FRM, IMP) were evaluated at ground level and the DRN at 10 m above ground level. Although the drone was only flown for 10 min per interval in our campaigns, we evaluated its performance over 45-min intervals to account for potential future battery or tethering improvements. The 100-grain threshold corresponds to different effective concentrations for each sampler type: 3.7 pollen/m$^3$ for ED, 133 pollen/m$^3$ for FRM, and 3.7$\times 10^3$ pollen/m$^3$ for IMP and DRN.

Figure 9 shows that, to collect at least 100 pollen grains, the ED samplers require 1.5 orders of magnitude less emission rate than the FRM and 3 orders of magnitude less than the IMP, across all distances from the field. This advantage allows ED samplers to be placed significantly farther from the field compared to IMP and FRM samplers. The DRN requires an even greater emission rate, due to lower concentrations at 10 m altitude. However, at approximately 200 m downwind, the DRN and IMP detection capabilities converge, as vertical concentration gradients become less pronounced further from the source.

The gray vertical lines in Fig. 9 represent computed emission rate values at each ED sampler during our field campaign, which are also shown in Fig. 7. These results indicate that only the ED samplers had a reasonable chance of collecting at least 100 particles during some sampling intervals. The remaining samplers were largely ineffective at detecting airborne pollen given the small emission rates observed in this study, even at close proximity to the source field. For a larger source, with an emission rate of 10$^6$ pollen/s (approximately 100 times larger than ours), the feasible placement of samplers would improve substantially. Under such conditions, impingers could be placed up to 10 m from the field, the FRM samplers could be placed up to 100 m from the field, and ED samplers could be placed up to 200 m away. These adjustments would allow each sampler type to collect at least 100 pollen grains within a 45-min sampling interval, enhancing measurement reliability and reducing uncertainty.

## Discussion

In these field experiments, airborne pollen from two different strains of OFP-expressing switchgrass plants was captured and analyzed using the method described in Rice et al. (2013). The PSYBIN1a plants exhibited strong fluorescence compared to wild-type pollen, whereas the pANIC10A pollen was difficult to distinguish by fluorescence alone. However, since the PSYBIN1a plants did not release sufficient pollen during any of the field experiments, OFP expression was not used for pollen identification in this study. If we had successfully captured PSYBIN1a pollen from the younger field, it would have expedited the counting and sampling process. Despite this limitation, the study serves as a proof-of-concept that fluorescence tagging can be a valuable tool for pollen tracking. Some level of autofluorescence was expected in WT pollen, as pollen walls naturally contain autofluorescent compounds including sporopollenin, flavonoids, and other phenolic constituents (Donaldson, 2020). This endogenous fluorescence likely contributed to the background signal observed under OFP excitation conditions and reduced discrimination between WT pollen and the weaker pANIC10A signal. These findings highlight the importance of fluorophore selection, promoter strength, and excitation/emission filter optimization for future fluorescence-based pollen tracking studies. Though prior studies have tracked the movement of pollen in the atmosphere (Aylor & Flesch, 2001; Aylor et al., 2006, 2003; Frisk et al., 2023; Millwood et al., 2017), to our knowledge, this is the first detailed study to incorporate aerial and ground-based volumetric samplers to track the movement of GE pollen from a known source. Understanding switchgrass gene flow is particularly relevant as biofuel production increases, helping to mitigate ecological risks posed by invasive strains and unintended cross-pollination between transgenic varieties (Kwit & Stewart, 2012). Fluorescent tagging presents a unique opportunity to trace GE pollen dispersal to wild and non-GE populations, potentially over long distances, as it enables direct identification of transgenic pollen without receptor plants and progeny analysis (Millwood et al., 2017). Additionally, fluorescence-tagged pollen could facilitate rapid and automated counting using instruments such as the Helmut Hund BAA500, Plair Rapid-E, Swisens Poleno, or WIBS-4 (Buters et al., 2024; O’Connor et al., 2014), which is of particular interest for allergen monitoring.

During the final campaign on August 2–3, 2022, a distinct diurnal pattern emerged in both measured pollen concentrations and computed pollen emission rate. Pollen emission rate increased steadily after the first sampling interval at 10:00, peaked at 14:00 on both days, and declined during the final sampling interval at 15:00. This diurnal pattern was consistent across both days and correlated with increasing wind velocity and temperature, as well as decreasing relative humidity. Such diurnal pollen release patterns are common in wind-pollinated species, where anther dehiscence is driven by drying conditions such as low humidity and rising temperatures (Sofiev & Bergmann, 2012). Similar trends have been observed in previous switchgrass field studies, where peak pollen concentrations occurred in the late morning and early afternoon, followed by a decline around 15:00 (Auer et al., 2016). Comparable findings in maize have linked pollen release patterns to increasing vapor pressure deficit (Jarosz et al., 2005) and decreasing relative humidity combined with rising wind velocity (Marceau et al., 2011). This information can be used to better predict peak allergen concentrations and improve the accuracy of large-scale air pollution models. Similarly, these diurnal emission patterns could inform gene flow risk windows for transgenic crops, allowing regulators to identify the time periods during which pollen-mediated gene flow to wild populations is most likely.

Throughout all sampling intervals and field campaigns, we observed very low-wind velocities (< 2 m/s) and frequent shifts in wind direction. Under these meandering wind conditions, particle dispersal is primarily controlled by wind direction shifts rather than turbulence (Anfossi et al., 2005; Vickers et al., 2008). Standard dispersal models that assume a dominant downwind direction fail to account for this effect, often producing overly narrow plumes that underestimate lateral spread. This limitation is particularly characteristic of LS models (Anfossi et al., 1990; Jarosz et al., 2005) and Gaussian plume models (Sagendorf & Dickson, 1974) which require a single downwind direction. Even more advanced modeling approaches, such as Large Eddy Simulations, do not fully incorporate changing wind directions (Chamecki et al., 2009). To address this, we reduced the averaging window for wind statistics from 45 to 5 and 1 min, and then ran dispersal models for each of these smaller intervals and combined the resulting plumes. This approach dramatically improved the fit between modeled and measured concentrations, enhancing emission rate estimates. Similar techniques have been applied in Gaussian plume modeling with 2-min intervals, yielding significantly better agreement with measured data (Sagendorf & Dickson, 1974). Anfossi et al. (1990) also emphasized the importance of using short averaging windows for dispersal modeling, recommending intervals of only a few minutes. A maize dispersal study similarly attributed discrepancies between measured and modeled concentrations to wind direction variability and assumptions of a dominant wind direction (Jarosz et al., 2005). Accurately modeling dispersal under these common low-wind conditions is particularly relevant for predicting gene flow to wild switchgrass populations, where even small amounts of viable pollen at distant locations can result in cross-pollination (Kwit & Stewart, 2012; Millwood et al., 2017). Future large-scale pollen forecasting and bio-confinement strategies should consider meandering wind conditions, which are not currently accounted for in large-scale models (Chamecki et al., 2009).

The highest concentration measurements in this study came from the high-volume ED samplers. The pollen source size—100 plants releasing pollen—was exceedingly small compared to previous dispersal experiments in switchgrass, which involved 3200 plants (Auer et al., 2016), as well as studies in maize (Aylor et al., 2006; Jarosz et al., 2005; Marceau et al., 2011). High-volume ED samplers performed best under these small source conditions, capturing spatial variations in concentration and resolved diurnal emission patterns at hourly timescales. To the best of our knowledge, this is the first pollen-trapping field study to utilize these ED samplers. Their volumetric flow rate of 600 L/min is 60 times greater than that of the commonly used 10 L/min Hirst-type samplers, similar to the 16.7 L/min FRM sampler, which did not collect enough pollen in this study. As the ED samplers were originally designed for educational purposes, they are inexpensive and lack pre-programming and other advanced features found in commercial volumetric samplers. Their simplicity and affordability, however, make them easily deployable. For GE dispersal studies, ED samplers offer a marked improvement over the passive pollen traps used in previous switchgrass gene flow experiments (Millwood et al., 2017). Their high intake rate enables detection at lower concentrations and greater distances, and their time-resolved volumetric measurements can drive dispersal models that predict gene flow risk to wild switchgrass populations beyond the sampler network.

A novel impinger-type particle sampler (IMP and DRN) was used in this study to collect pollen, marking the first application of this integrated system for pollen tracking. While previously employed for airborne microbial sampling (Bilyeu et al., 2022; Powers et al., 2018), this study extends its use to pollen dispersal. The IMP and DRN samplers successfully collected pollen in the field, demonstrating their feasibility for tracking pollen movement. However, due to the small source size, limited pollen production, and the relatively low sampling rate of 0.6 L/min, the collected pollen quantities were insufficient for reliable concentration estimates. The differing flow rates between the ED and IMP samplers further complicate direct comparisons, as impinger samplers inherently capture fewer particles at high concentrations due to their small intake volumes. The IMP system would likely perform more effectively when sampling from much larger sources, at least 100 times the size of the field used in our study (Fig. 9). Similarly, the drone-mounted sampler, operating at 10 m above ground level, would require a significantly larger pollen source for effective deployment at further distances and altitudes. Nevertheless, the drone platform remains a valuable tool for aerobiological research, offering future opportunities for prolonged and spatially resolved sampling, particularly when paired with higher-volume sampling technologies, including those incorporating filter-based collection systems. Moreover, impinger samplers, which preserve particles in liquid, could prove especially useful for future viability and molecular studies.

A primary constraint in regulated transgenic pollen dispersal studies is the feasible scale of the pollen source. From an agricultural perspective, a 100-plant source is small relative to other agricultural dispersal studies. However, in the context of permitted flowering GE pollen dispersal, it is substantial because it requires specialized propagation, approved experimental sites, and a stringent regulatory permitting process. Our experimental design therefore prioritized a small but well-contained OFP-tagged source, and we structured the sampling campaign to extract the most robust insights that this rare setup could support. The value of the present study is therefore not that it reproduces a large agricultural pollen release, but that it establishes what is measurable and how to model it under realistic constraints that are intrinsic to transgenic field experimentation.

With more resources, the natural next step would be a scaled-up version of this same experiment focused on validation: a large, well-established source containing only PSYBIN1a to maximize the OFP signal, multi-year sampling on the same dates and times to quantify repeatability of diurnal emission patterns, and co-located Hirst-type gold-standard samplers deployed alongside the novel samplers to provide independent concentration estimates for model validation and rigorous sampler inter-comparison. A substantially larger source would allow meaningful comparisons between low- and high-volume samplers, with one set used to estimate the particle release rate and another for validating modeled concentrations. It would also enable more effective use of impinger-type samplers (IMP and DRN), which could preserve pollen for downstream viability studies, although approved experimental site requirements for transgenic work would continue to pose limits for long-distance tracking.

Together, the methods demonstrated in this study—high-volume sampling, fluorescence-based source attribution, emission rate estimation, and improved low-wind dispersal modeling—provide a framework for applications requiring airborne pollen tracking, including monitoring GE pollen transport to wild and non-GE populations, assessing cross-pollination risk between fields, and improving the temporal resolution of allergen forecasting. This integrated approach is also transferable to other wind-pollinated crops of interest. For instance, hemp is known to produce copious amounts of pollen capable of long-distance dispersal, and its monitoring is increasingly relevant (Nimmala et al., 2024). If transgenic hemp lines become available, similar fluorescence-based tracking methods could be applied to study their pollen movement and gene flow in detail.

## Conclusions

Three field campaigns were conducted to measure pollen concentrations around a small field of genetically modified switchgrass, utilizing both traditional and novel sampler types. The experiments also included a drone-mounted sampler, demonstrating the feasibility of airborne pollen sampling at 10 m above the field as a proof-of-concept. Despite the exceedingly small source size, the high-volume ED samplers successfully collected sufficient pollen to analyze spatial variations in concentration and identify diurnal release patterns. This study evaluated the effectiveness of different sampler types for pollen collection under varying conditions. Among the three field campaigns, only the final campaign on August 2–3, 2022, produced sufficient concentration data for detailed analysis and modeling. During this campaign, a clear diurnal pattern was observed in the pollen concentration and, consequently, in the calculated emission rate. Persistent low-wind meandering conditions were recorded throughout the campaign, and reducing the averaging window for simulations significantly improved pollen emission rate estimations by better incorporating shifting wind directions. This study highlights the potential for drone-based pollen sampling and fluorescence-based GE pollen tracking. The findings provide insight into the effectiveness of different sensor types with respect to source strength and sampling distance, advancing the understanding of pollen dispersal dynamics and measurement techniques. These results have important implications for allergen monitoring, cross-pollination risk assessment, and broader bioaerosol surveillance strategies.

## Data Availability

All sampling data, modeling code, and simulation results underlying this manuscript are made available on the Virginia Tech Data Repository at https://doi.org/10.7294/25733604.
